# Feasibility of virtual low-cost quantitative continuous measurement of movements in the extremities of people with Parkinson’s disease

**DOI:** 10.1016/j.mex.2023.102230

**Published:** 2023-06-02

**Authors:** Abdelwahab Elshourbagy, Mennatullah Mohamed Eltaras, Hassan Abdalshafy, Samrah Javed, Ahmed Omar Sadaney, Timothy Patrick Harrigan, Kelly Alexander Mills, Manuel Enrique Hernandez, James Robert Brašić

**Affiliations:** a Misr University for Science and Technology, Al Motamayez District-6th of October, Giza Governorate 3236101, Egypt; b Faculty of Medicine for Girls, Al-Azhar University, Cairo Governorate 4434003, Egypt; c Faculty of Medicine, Cairo University, Giza Governorate 12613, Egypt; d Jinnah Sindh Medical University, Karachi, Sindh 75510, Pakistan; e Research and Exploratory Development, Applied Physics Laboratory, The Johns Hopkins University, Laurel, MD 20723, United States; f Department of Neurology, The Johns Hopkins University School of Medicine, Baltimore, MD 21205, United States; g Carle Health College of Medicine, University of Illinois at Urbana-Champaign, Urbana, IL 61801, United States; h Section of High-Resolution Brain Positron Emission Tomography Imaging, Division of Nuclear Medicine and Molecular Imaging, The Russell H. Morgan Department of Radiology and Radiological Science, The Johns Hopkins University School of Medicine, Baltimore, MD 21205, United States

**Keywords:** Accelerometer, Experimental error, Motor assessment, Movement disorders, Online, Rating scales, Structured interviews, Trained raters, Test-retest reliability, Visual observation, Virtual low-cost quantitative continuous measurement of movements in the extremities of people with Parkinson’s disease.

## Abstract

A low-cost quantitative continuous measurement of movements in the extremities of people with Parkinson‘s disease, a structured motor assessment administered by a trained examiner to a patient physically present in the same room, utilizes sensors to generate output to facilitate the evaluation of the patient. However, motor assessments with the patient and the examiner in the same room may not be feasible due to distances between the patient and the examiner and the risk of transmission of infections between the patient and the examiner. Therefore, we propose a protocol for the remote assessment by examiners in different locations of both (A) videos of patients recorded during in-person motor assessments and (B) live virtual assessments of patients in different locations from examiners. The proposed procedure provides a framework for providers, investigators, and patients in vastly diverse locations to conduct optimal motor assessments required to develop treatment plans utilizing precision medicine tailored to the specific needs of each individual patient. The proposed protocol generates the foundation for providers to remotely perform structured motor assessments necessary for optimal diagnosis and treatment of people with Parkinson's disease and related conditions.

## Graphical abstract

**Figure:**
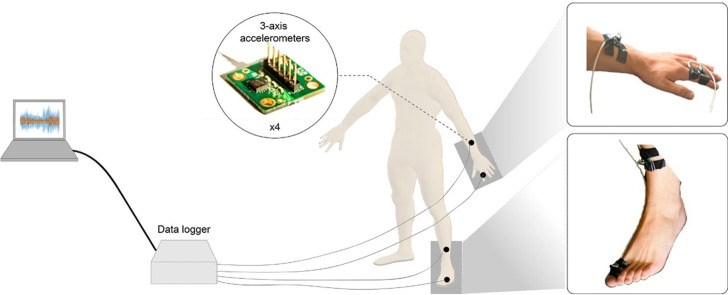
Schematic diagram of procedure to generate output from accelerometers on the extremities to record on a data logger for transmission to a laptop computer for conversion to signals for analysis 7] (Courtesy of Jenny-Ann Phan, MD, PhD).

Specifications table

**Table:** 

Subject area:	Medicine and Dentistry
More specific subject area:	Neurology
Name of your protocol:	Virtual low-cost quantitative continuous measurement of movements in the extremities of people with Parkinson‘s disease
Reagents/tools:	Zoom, Version 5.12.9 (5692), [software] (2023). Zoom Video Communications, Inc., https://zoom.us
Experimental design:	This protocol provides a method for trained raters at different locations to simultaneously score videos and real-time administrations of a low-cost quantitative continuous measurement of movements in the extremities of people with Parkinson‘s disease, a structured motor assessment with instrumentation. This procedure provides the means for remote raters to perform assessments online after the testing protocol has been completed or while the protocol is administered to a participant in a different location.
Trial registration:	Not applicable
Ethics:	The work was carried out in accordance with the recommendations of the World Medical Association in the WMA Declaration of Helsinki - Ethical Principles for Medical Research Involving Human Subjects [1] and the Recommendations for the Conduct, Reporting, Editing, and Publication of Scholarly Work in Medical Journals [2]. Written informed consent was obtained from all participants utilizing a protocol (IRB00110166) approved by Johns Hopkins Medical Institutions Institutional Review Board, Baltimore, Maryland, USA.
Value of the Protocol:	• Inexpensive accelerometers are attached to the upper and lower extremities of patients with Parkinson's disease and related conditions to generate a continuous, three-dimensional recorded representation of movements occurring while performing tasks to characterize the deficits of Parkinson's disease. • Movements of the procedure are rated by trained examiners (A) in real-time in the same room, (B) in real-time in different locations, and (C) after the testing sessions by videotapes. • This protocol provides the means for experts in multiple locations to remotely administer and score structured motor assessments of participants in underserved locations around the world.

## Description of protocol

### Rationale

Parkinson's disease, the second most common neurodegenerative disease, afflicts 1% of men and women [3] aged 60 and over and afflicts more individuals with increasing age [4]. The prevalence of Parkinson's disease is increasing around the world [5]. Structured interviews and examinations of participants to assess motor impairments common in people with Parkinson's disease were developed to be performed in person to utilize the full capability of human observation by trained examiners [6], [7], [8]. Additionally, the identification of tremors of people with Parkinson's disease has been facilitated by the application of automatic intelligence to video recordings [9]. However, a comprehensive review of the vast spectrum of computer-assisted technologies [10] and wearable sensors [11] that have been developed to facilitate the diagnosis and treatment of Parkinson's disease is beyond the scope of this article.

Since people who develop Parkinson's disease may exhibit motor symptoms up to 3 years before they receive the diagnosis of Parkinson's disease [12], simple procedures to identify people with Parkinson's disease and related conditions in underserved locations through online assessments are needed. Additionally, risks of infections, lack of transportation, and other environmental influences may prevent the conduct of live ratings. Therefore, we seek to develop simple inexpensive methodology for trained raters in different locations to simultaneously rate videos and live performances of structured motor assessments of individuals and to immediately generate a consensus rating of the output. For these reasons we have developed a protocol for conducting motor assessments online using Zoom [13]. We seek to share our proposed protocol for the use with underserved populations by colleagues around the world.

### Guidelines for optimal remote evaluation of videotaped motor assessments conducted in person

Since statistical procedures assume that the underlying populations are independent and identically distributed [14], data that is generated through similar procedures is crucial to the development of results suitable for meaningful analysis. The generation of data that is independently and identically distributed is facilitated by assessments conducted by the same trained rater with every participant in the same chair with arms without wheels in the same room at the same temperature at the same time of day. Videotapes of the testing procedures provide the foundations to assess the live ratings of the trained examiner and the recorded images.

### Construction of protocol videotapes

A low-cost quantitative continuous measurement of movements in the extremities of people with Parkinson‘s disease [7,8] was performed in person by an examiner certified in the Movement Disorder Society-sponsored revision of the Unified Parkinson's Disease Rating Scale (MDS-UPDRS) [6] on cohorts of participants with Parkinson's disease (*N* = 20), multiple system atrophy [*N* = 1], and typical development [*N* = 8] with the assistance of a technologist who recorded and processed the output of the accelerometers and videotaped the participant. Accelerometers were taped to the upper and lower extremities of participants before the participants were asked to perform twelve tasks modified from the MDS-UPDRS [[6], [7]]. Each task (Table 1) of a low-cost quantitative continuous measurement of movements in the extremities of people with Parkinson‘s disease [7,8] was administered to participants and the score recorded immediately by the examiner [7,8,15]. The procedure was performed to facilitate the identification of the severity of the typical impairments experienced by people with Parkinson's disease. The output provides the bases for clinicians to assess the current functioning of people with Parkinson's disease to foster the development of a treatment plan tailored to the specific needs of each individual.

**Table 1:** Categories and descriptions of tasks assessed by a low-cost quantitative continuous measurement of movements in the extremities of people with Parkinson's disease [7].

**Rest tremor**

3.17 Rest tremor amplitude upper limb (RTU)
• The participant is instructed to sit quietly in a straight-back chair with hands on the arms of the chair and feet comfortably on the floor for three minutes.
3.17 Rest tremor amplitude upper limb counting (RTUC)
• The participant is instructed to sit quietly in a straight-back chair with hands on the arms of the chair and feet comfortably on the floor and to count out loud backwards from 30.
3.17 Rest tremor amplitude lower limb (RTL)
• The participant is instructed to sit quietly in a straight-back chair with hands on the arms of the chair and feet comfortably on the floor for three minutes.
3.17 Rest tremor amplitude lower limb counting (RTLC)
• The participant is instructed to sit quietly in a straight-back chair with hands on the arms of the chair and feet comfortably on the floor and to count out loud backwards from 30

**Postural tremor**

3.15 Postural tremor of the hands (PT)
• The participant is asked to separately stretch each hand out in front of the body with palms down and fingers comfortably separated so that they do not touch each other for ten seconds.

**Repetitive movements**

3.4 Finger tapping (FT)
• The participant is instructed to tap the index finger to the thumb as fast and fully as possible for 60 repetitions.
3.5 Hand movements (HM)
• The participant is instructed to make a tight fist and to open and close the fist as fast and fully as possible for 60 repetitions.
3.6 Pronation-supination movements of the hands (PS)
• The participant is instructed to stretch each arm with palm down and to turn the palm up and down as fast and fully as possible for 60 repetitions.
3.7 Toe tapping (TT)
• The participant is instructed to place each foot comfortably on the floor and to keep the heel on the floor while tapping the foot up and down as fast and fully as possible for 60 repetitions.
3.8 Leg agility (LA)
• The participant is instructed to place each foot comfortably on the floor and to raise the foot up and down as fast and fully as possible for 60 repetitions.

**Change of position**

3.9 Arising from chair upper limbs (ACU)
• The participant is instructed to cross arms on chest and to stand up from sitting.
3.9 Arising from chair lower limbs (ACL)

A video camera on a tripod were utilized to record the assessments. The orientation of the video camera was consistently directly facing the participant. The video camera was placed in the same position at the edge of the table in the conference room used for the ratings. The participant was consistently placed in the same position in the conference room for the rating sessions. The video camera was approximately three feet from the participant for all ratings. The recording files were copied to a computer for storage for future analysis. The original video recordings contained uninterrupted files of all the discussions among the examiner, the participant, and the technologist before and after each task. The original videos were entered by an investigator in North America (JRB) into folders on Google Drive.. The original videos were then downloaded by an investigator in Africa (AE) from the folders on Google Drive to a computer for editing. In order to construct the videos for a compact manner suitable for efficient rating, the investigator in Africa [AE] constructed individual video recordings of each individual task with a second or so before and after each task [16,26,27]. A typical video editing session took 30 to 60 min. The process of uploading each original file to Google Drive, downloading each original file from Google Drive, and editing videos with only the individual tasks took a day or two for the processing of a single video session for a single participant. The remote rating of the edited videos of a single assessment of a single participant and the consensus conference took one to two hours.

The rater and the participant had no communication after the original videos were obtained with the exception that the healthy participants for this study were all co-authors who continued to collaborate. One of the healthy men (JRB) also participated in the construction and rating of the edited videos. There was no online contact between raters and other participants at the time of the ratings of the videos. The original clinical scores obtained by the trained examiner and the instrumentation signals and their transforms have been published [15], [16], [17], [18], [19], [20], [21], [22], [23]. We seek now to develop a protocol for raters all in different locations to rate the videotapes to share with colleagues around the world.

A preliminary step before rating sessions was to prepare the videos in a format convenient for efficient rating sessions. The original videotapes were uninterrupted recordings of all activities of the participants and raters. The original videotapes contained the intermissions between the conduct of the actual tasks when the technologist processed and recorded the output of the accelerometers and set up the computer to record the next task. There typically were several minutes on the videotape between the actual tasks of the protocol. Since those intermissions between tasks are not rated, they were edited out of the videotapes provided to the raters for each rating session. The original video segments are too large to be sent as email attachments, so they are placed on folders on the iCloud for storage and transmission to team members. Uploading a video of 30 to 60 min may take several hours. Downloading the videos from the iCloud is tedious and time-consuming. Technical problems may interfere with the quick and effective transmission of the videos. An examiner in North America who conducted original in person motor assessments and participated as a participant with typical development (JRB) and an examiner in Africa (AE) met online to view the original videotapes lasting 30 to 60 min to construct video clips of each specific motor task with a second or so before the start of the task and a second or so after the completion of the task to generate separate video clips of each task lasting approximately 15 to approximately 180 s. The initial attempts to construct video clips suitable for online rating were halted after edited files from North America placed on Mendeley Data did not play with sound in North America. Limitations of the computers and Internet systems likely prevented the accurate transmission and playing of the documents. The edited files could be placed on Mendeley Data from Africa and could be send and heard when played in Africa. In order to proceed with the project for international publication, the investigator in Africa (AE) took on the role of corresponding author to place the video clips and related materials on Mendeley Data [16,26,27]. The investigators in North America (JRB) and Africa (AE) met regularly to check the accuracy of the sights and sounds of each video clip placed on Mendeley Data [16,26,27]. The investigators proceeded with the construction of the placement of videos and related materials on Mendeley Data with the hope that they could be accessed by colleagues for future studies.

Rating of the videos of the original assessments was conducted by a team of trained raters certified in the MDS-UPDRS [6] who were all in different physical locations. For this preliminary study to demonstrate the feasibility of online video rating by raters at different international locations, six raters certified in the MDS-UPDRS [6] were recruited. The ratings were performed by the six raters including the two raters (JRB and AE) who took part in the earlier aspects of the study. JRB and AE were not blind to the content of the video clips because they had participated in the earlier parts of the study. The other raters had no knowledge of the content of the videos. The other four raters were provided no information about the participants in the videos. The videos were presented to all raters for the rating sessions with only the participant identification (ID) number. The diagnosis, age, and sex of the participants in the videos was not provided to the raters during the rating process. Therefore, the other four raters were blind to the clinical and demographic data about the participants. Because this was a preliminary study of feasibility of the protocol, the outcome is reported without attaining statistical significance in the analysis. We had sought to obtain at least ten blind raters for the project. However, we proceeded to assess feasilibity as reported in this article with only six raters. For this preliminary assessment of feasibility, we encountered much missing data (Supplementary Table 1). Supplementary Table 1 contains the independent scores (0,1,2,3,4) of the six identified independent raters of videos of the motor tasks of a low-cost quantitative continuous measurement of movements in the extremities of people with Parkinson's disease [7] of five participants and the corresponding percentage agreements for consensus scores represented by %. Missing data is represented by a period (.) in Supplementary Table 1. The headings include the number and abbreviation of each task. Appendix 1 [7] in Supplementary data contains the coding form with the numbers and names of each task utilized by raters for each rating session.

Although we strived to conduct rating sessions at times convenient for all raters, the participation of all raters for all sessions did not happen. There were many internet disconnections so that only some ratings were obtained from some raters for some items. We proceeded with the project despite the small numbers of raters and the missing data in order to identify the strengths and weaknesses for further development of our procedure. We sought to establish that this procedure is feasible for a team of diverse raters each in different locations. A power analysis would facilitate the determination of the number of raters for a definitive statistical analysis for future investigations. Due to many technical discrepancies (lack of sound and blurred images) observed by the original investigator (JRB), the other investigator (AE) hosted all joint sessions and presented the videos on his monitor to screen share with the other raters. After the twelve tasks of a low-cost quantitative continuous measurement of movements in the extremities of people with Parkinson‘s disease [7,8] were edited as separate video clips, the two examiners who had seen the videos (AE and JRB) recruited a team of raters who had attained certification in the MDS-UPDRS [6] to rate each video simultaneously. Altogether there were six raters, the two raters who had participated in the design and conduct of the study (AE and JRB) and the four raters who had never participated in the study. For each rating session the investigator (AE) provided only the participant indentification (ID) number without any clinical or demographic information. The four recruited raters were blind to the identity of the participants in the videos while the two raters who participated In the design and conduct of the study were not blind to the identify of the participants. Rating sessions were scheduled when all raters could attend; however, there was missing data for each rater due to internet disconnections.

Videotape ratings are facilitated by performing the task in a manner such that all raters would have similar experiences. In the past when raters could readily gather together, trained raters were seated in a room with a similar view of a monitor to observe videotapes jointly. Raters were allowed to see the recordings once and required to make ratings independently [[24], [25]]. Immediately after completing all ratings without consultation with anyone else, the raters conducted a consensus conference to discuss the findings to attain a scored agreed by all raters.

The onset of severe respiratory infections led to the prohibition of gatherings together in person. Therefore, this protocol was developed to accommodate a means to provide a uniform rating experience for independent raters using monitors in different locations. Although raters are not physically present in the same room, they are provided one viewing jointly on their own monitors. The raters then independently complete their rating immediately after being shown each task. After all ratings are completed, the raters send their scores to the investigator (AE) who then conducts a consensus conference with all raters to then discuss each task with each other for the first time. The team then seeks to agree on a score for each item.

### Method feasibility

Rating sessions were hosted by the investigator (AE) who presented the clips [16,26,27] for viewing and who provided to the raters only the code number of the participant without name, age, sex, diagnosis, or other identifying characteristics. The investigator (AE) stated the code number of the participant to be entered by the raters on the score sheet (See Appendix 1 [7] in Supplementary data). The investigator (AE) then stated the number and the name of each task [7] so that the raters could read the scoring instructions for that task. The raters were provided no other information about each participant. No demographic or clinical data about participants was provided to the raters. The investigator (AE) then played the relevant video clip once on his monitor screen shared with the raters. The investigator (AE) then asked each rater to score the tasks independently without discussion with anyone else. The investigator (AE) asked all raters to apply the scoring instructions to all segments of the video clips, even though the video segments were much longer than the those for the MDS-UPDRS [6]. The duration of repetitive items included up to 60 or so repetitions in order to generate enough signals suitable for analysis [7]. The raters were asked to apply the scoring criteria (See Appendix 1 [7] in Supplementary materials) as best they could to the observed video segments, despite blurring and delays in the videos resulting from technical limitations in the cameras, computers, monitors, and Internet disconnections. After all of the 12 items [7] were scored independently by the raters, the investigator (AE) asked all raters to send him electronic copies of their score sheets. Then the investigator (AE) conducted a consensus conference with all raters to attain agreement for the score for each item. If the raters did not agree, then a period was entered as the consensus score to indicate that was missing.

To assess interrater reliability among raters, percent agreement was calculated for each item (Supplementary Table 1). The agreement attained among trained raters viewing videos remotely is comparable to the agreement attained in other studies among trained raters viewing videos in person in the same room [[24], [25]]. In order to improve the agreement among raters, consensus conferences were conducted immediately after each rating session. Each rater stated their independent score. Then, the raters discussed the basis for their scores. The discussions led to the agreement on a consensus score after considering the opinions of other raters. Therefore, a consensus conference was conducted to provide an overall estimate of the viewpoints of the team of raters.

The consensus scores of six raters for the test rating sessions of five participants aged 66.2 ± 9.2 (55, 76) years including four men (three healthy controls with typical development and one participant with Parkinson's disease) and two people (one man and one woman) with Parkinson's disease (PD) are presented in Table 2. Altogether 110 consensus conferences scores were condcuted attained by the six raters of the 22 video clips of the protocol conducted on five participants. Raters on three continents agreed on 28 scores of 0, 14 scores of 1, 4 scores of 2, and 7 scores of 3. The raters did not agree on scores for 57 video clips indicated by periods (.) on Table 2. Table 2 contains the item numbers corresponding to the original items in the MDS-UPDRS [6] and the low-cost quantitative continuous measurement of movements in the extremities of people with Parkinson's disease [7]. Interruptions and blurring of video images presented challenges for rating using the techniques of visual observation for live rating. Internet disconnections prevented some ratings during the experimental sessions. Virtual assessment of motor assessment videos presented on the monitor of an investigator (AE) rated by examiners with different monitors in different locations can be accomplished. Agreement for tasks with stationary positions of the extremities was better than for repetitive movements.

**Table 2:** Demographic traits and consensus scores of six independent raters of videos of motor tasks of five participants.

Age	PD	Male	Ht	Wt	3.17 RTUR	3.17 RTUL	3.17 RTUCR	3.17 RTUCL	3.15 PTR	3.15 PTL	3.4 FTR	3.4 FTL	3.5 HMR	3.5 HML	3.6 PSR	3.6 PSL	3.9 ACU
76	1	1	72	178	0	.	0		1	.	.	.		.	.		1
70	0	1	61	122	0	0	0	0	.	.		.		.	.	.	0
72	1	0	64	177	1	1	1	1	1	1	.	3	.	.	.	.	0
58	0	1	71	215	0	0	0	0	2	2	.		3	3	3	3	0
55	0	1	67	159	.	.	.	.	.	.	1	.	.	.	1	1	.

Age	3.17 RTLR	3.17 RTLL	3.17 RTLCR	3.17 RTLCL	3.7 TTR	3.7 TTL	3.8 LAR	3.8 LAL	3.9 ACL
76	.	.	0	.			.	.	1
70	0	0	0	0	.	.	2	2	0
72	1	0	1	0	.	.		.	0
58	0	0	0	0	3	3	.	.	0
55	.	.	.	.	.	.	.	.	.

Age: Age in years; PD: Parkinson's disease 1 = present, 0 = absent (healthy control with typical development); Male: Male 1 = present, 0 = absent (female); Ht: Height in inches; Wt: Weight in pounds; 3.17 RTUR: 3.17 Rest tremor amplitude upper limbs right; 3.17 RTUL: 3.17 Rest tremor amplitude upper limbs left; 3.17 RTUCR: 3.17 Rest tremor amplitude upper limbs right counting; 3.17 RTUCL: 3.17 Rest tremor amplitude upper limbs left counting; 3.15 PTR: 3.15 Postural tremor of the hands right; 3.15 PTL: 3.15 Postural tremor of the hands left; 3.4 FTR: 3.4 Finger tapping right; 3.4 FTL: 3.4 Finger tapping left; 3.5 HMR: 3.5 Hand movements right; 3.5 HML: 3.5 Hand movements left; 3.6 PSR: 3.6 Pronation-supination movements of the hands right; 3.6 PSL: 3.6 Pronation-supination movements of the hands left; 3.9 ACU: 3.9 Arising from chair upper limbs; 3.17 RTLR: 3.17 Rest tremor amplitude lower limbs right; 3.17 RTLL: 3.17 Rest tremor amplitude lower limbs left; 3.17 RTLCR: 3.17 Rest tremor amplitude lower limbs right counting; 3.17 RTLCL: 3.17 Rest tremor amplitude lower limbs left counting; 3.7 TTR: 3.7 Toe tapping right; 3.7 TTL: 3.7 Toe tapping left; 3.8 LAR: 3.8 Leg agility right; 3.8 LAL: 3.8 Leg agility left; 3.9 ACL: 3.9 Arising from chair lower limbs; period (.): absence of consensus agreement.

(McKay GN, Harrigan TP, Brasic JR. A low-cost quantitative continuous measurement of movements in the extremities of people with Parkinson's disease. MethodsX 2019; 6:169–189. https://doi.org/10.1016/j.mex.2018.12.017).

### Protocol for the remote evaluation of motor assessments conducted online

A low-cost quantitative continuous measurement of movements in the extremities of people with Parkinson‘s disease [7,8] was performed online by an investigator (AE) certified in the Movement Disorder Society-sponsored revision of the Unified Parkinson's Disease Rating Scale (MDS-UPDRS) [6] on a 70-year-old man with Parkinson's disease who had begun oral medications for Parkinson's disease. Sessions with the participant were conducted monthly to obtain a sequential record of his functioning during the course of his treatment. The investigator (AE), the participant, and all raters were connected online at separate computers in different locations. The investigator (AE) obtained information about the participant's height during the first session. For each session the investigator (AE) asked about the weight of the participant, all current medications, and the on or off effect of levodopa. The investigator (AE) asked the participant to provide permission to videotape the assessments to be shared with the team and to be presented at professional conferences and published in professional journals. If the participant did not agree to be videotaped, the session proceeded without a videorecording. If the participant agreed to be videotaped, the session was videotaped. The investigator (AE) administered the instructions and demonstrated the tasks [7,8]. When the participant was positioned so that the relevant components of the protocol could not be visualized by others, the participant was asked to position himself and his camera so that a clear image could be obtained on the monitor.

The investigator (AE) stated the identity of the participant to be entered by the raters on the score sheet (See Appendix 1 [7] in Supplementary data). The investigator (AE) then stated the number and the name of each task (See Appendix 1 [7] in Supplementary materials) so that the raters could read the scoring instructions for that task. The investigator (AE) presented the monitor of the participant to all raters while he administered each task to the participant. The raters saw the participant during the administration of each task to then score each task immediately after it was accomplished. The iinvestigator (AE) asked each rater to score the tasks independently without discussion with anyone else. The investigator (AE) asked all raters to apply the scoring instructions (See Appendix 1 [7] in Supplementary materials) to all tasks in the live rating sessions, even though the tasks were conducted for durations that were much longer than the instructions for the MDS-UPDRS [6]. The raters were asked to apply the scoring criteria as best as they could to the observed live tasks performed by the participant on their monitor, despite blurring of fine movement, delays in transmission to the computers of the raters, and Internet disconnections. After all of the twelve items were scored independently by the raters, the investigator (AE) asked all raters to send him electronic copies of their score sheets. The participant and the raters agreed to date and time of the next monthly rating session The participant was thanked for each session and was excused. Then the investigator (AE) conducted a consensus conference with all raters to attain agreement for the score for each item. If the raters did not agree, then a period was entered as the consensus score to indicate that consensus was missing. The online ratings are continuing so results are not yet available.

### Advantages

The proposed protocol provides the means for two experts in different locations can accomplish the extraction of the motor assessment tasks from original videos containing the extensive intermissions between the motor tasks that are irrelevant to the rating process. The protocol provides the means for experts to check each other to accomplish the construction of separate videos containing only the segments needed to rate each task.

The proposed protocol provides the framework for raters using different computers in different locations to independently and identically perform structured ratings of motor assessment videos coordinated by a coordinator utilizing a different computer in a different location. Since the trained raters conducted independent ratings of identical video screenings, the assumptions of probability theory and mathematical statistical analysis are attained [14]. All raters are shown the test video on shared screen of the investigator's monitor once, make independent scoring immediately after viewing the video, and transmit their scores to the investigator (AE) at the same time. The investigator (AE) then obtains discussion from the raters to agree on a consensus score for each task.

### Disadvantages

The original testing procedure by the participant, the examiner, and the technologist jointly in person in a room requires an hour or so to accomplish. One problem with the protocol is the need for the examiner to tape accelerometers on the extremities of the participants and to connect the accelerometers by wires to a data logger connected by wires to a laptop computer before administering the task to the participant. Another problem with the protocol is the need for the technologist to program the laptop to record the output from the accelerometers and then to process the output to generate and record signals for future analysis. Since the participant is connected by wires to a data logger, the movements of the participant are limited to raising and lowering extremities and standing up and down.

The original video files of 30 to 60 min are huge electronic files. They are too large to send as email attachments. The process to place then on folders in the iCloud may take several hours. Downloading the original files from the iCloud is time-consuming. Viewing the original videos to extract only the portions of the video with the motor task is tedious and time-consuming.

A further disadvantage of the protocol is the amount of time required for recording, uploading, editing, and sharing the video components. We have demonstrated that the protocol is feasible to conduct by experts certified in the MDS-UPDRS [6] in distant locations. This protocol provides the means to conduct complex assessments by a team of experts to generate similar output for comparison and contrast. Thus, the protocol establishes the framework for the precision and accuracy required for optimal clinical investigations. While an alternate protocol could utilize an expert at the original assessment site to record, edit, and share the videos, such a protocol is not feasible in underserved regions lacking experts with the required skills. Another alternate protocol could utilize a remote test expert administrator certified in the MDS-UPDRS [6] who views the live assessment conducted by test facilitator in the same room as the participant without editing, a protocol that we are currently developing ourselves. A further alternative could be the development of software to cue the test administrator to start and stop the video editing for each task to eliminate the need for editing the video.

### Videos

The videos were obtained at the time of the live ratings in person utilizing a videocamera on a tripod or a mobile phone on a tripod. The original videotapes were copied to be provided to members of the team. The videotapes were further copies to generate edited clips of the specific tasks for this study. The quality of the images deteriorated due to time, storage, and copying. There was blurring of images and sounds in the videos used for rating by teams.

### Online rating sessions

Online sessions were conducted with each person on a different electronic device in different locations. Each person experienced occasional interruptions of internet connections. The basic online program led to meetings ending after 40 min so there were intermittent disconnections. When individuals were disconnected, they were sent a new link by the investigator (AE) to connect to a new online meeting.

### Human visual observation

Drawbacks to the use of clinical ratings [6], [7], [8] include the reliance on real-time human vision to quantify small differences in motion and significant inter-rater variability due to inherent subjectivity in scoring the procedures. Rating tools are semi-quantitative by design, however, in addition to significant inter-rater variability, there is inherent subjectivity in administering these tools, which are not blinded in clinical settings.

### Future directions

#### Raters

Replication of the proposed protocol by colleagues independently at multiple centers will provide the foundation to confirm the usefulness of the procedure. To satisfy the assumption that the ratings are generated from populations that are identically and independently distributed, future studies will include teams of raters who complete ratings for all assessment in exactly the same manner with an adequate sample size to apply statistical analysis to yield significant findings [28]. A power analysis based on the initial findings of our preliminary study of the feasibility of the procedure [29,30] would be limited by the extensive missing data (Supplementary Table 1). A power analysis would facilitate the determination of the number of raters for a definitive statistical analysis for future investigations. Reliability can be studied by the use of the kappa statistic [[30], [31]]. We have used percent agreement [32] to obtain a rough estimate of the reliability of our preliminary study of feasibility [29,30].

#### Wireless sensors

The construction of wireless wearable sensors will be crucial to the development of a protocol suitable for marketing for practitioners. The current protocol is limited by the restrictions imposed on the movements of the participants by the connections of the instrumentation on the participants to the recording equipment. The accelerometers fell off participants requiring taping to reposition them. Also, the movement of the participant was restricted to standing in place due to the limited lengths of the cords. Wireless sensors will allow walking to be included in the assessments. Additionally wireless sensors will facilitate continuous evaluation of participants in the clinic and throughout the day conducting social, occupational, and educational activities.

#### Videos

Optimal videocameras will be used to generate top-notch images. Three videocameras will be used to provide clear images of participants in all three directions.

#### Online rating sessions

Optimal online monitoring will be used for meetings. Raters may be allowed to view videos at their convenience to facilitate optimal circumstances. Raters may be allowed to view videos as many times as they like in order to determine their optimal ratings. Raters may be allowed to view the videos at a slow speed to facilitate detecting rapid movements. Raters may even see the videos frame by frame in order to count repetitions and interruptions and to measure changes in rate and rhythm.

Future investigations will be enhanced by including more participants with and without Parkinson's disease and more trained raters. Using optimal cameras and monitors will enhance the quality of images presented to raters. Use of a toggle switch to examine individual frames will help rating by visual observation. Colleagues around the world can utilize this virtual protocol to obtain state-of-the-art motor assessments of people with PD and other conditions.

### Methods

Several novel techniques provide the foundations to utilize to develop and enhance this protocol. We seek to collaborate with those who have developed innovations to enhance with our proposed protocol. We are developing this protocol with groundbreaking related techniques [10,[15], [16], [17], [18], [19], [20], [21], [22], [23], [26], [27],^29^,^30^,[33], [34], [35], [36], [37], [38], [39], [40], [41], [42], [43], [44], [45], [46]]. In particular, we are exploring ways to utilize computers and other technologies to construct methods to efficiently and effectively measure movements in clinical settings [10,[15], [16], [17], [18], [19], [20], [21], [22], [23], [26], [27],^29^,[33], [34], [35], [36], [37], [38], [39], [40], [41], [42], [43], [44], [45], [46]], generate signals and transforms to generate signatures of Parkinson's disease and related conditions [[7], [8], [9], [10], [11], [12],[15], [16], [17], [18], [19], [20], [21], [22], [23], [24], [25], [26], [27],^29^,^30^], and employ automatic intelligence to provide diagnoses for immediate use by providers [33,[43], [44], [45], [46]].

#### Rating by automatic intelligence

Automatic intelligence may improve the scoring of the MDS-UPDRS [6,33,[43], [44], [45], [46]]; however, machine learning does not yet match the accuracy of trained human raters [44]. A comprehensive review of the technology to rate motor assessments is beyond the scope of this article. While the use of machine learning removes the limitations of human vision, it also removes the judgment of experienced human raters. The current protocol has the advantage of using experienced human raters certified in the MDS-UPDRS. Compentent clinicians who evaluate and treat patients with movement disorders require assessments by experienced humans to detect the subtleties that distinguish the various movement disorders. Clinicians are unwilling to base the diagnosis and treatment of people solely on the output of automatic intelligence without interpretation by an experienced human. We ourselves are developing the use of neural networks to evaluate output of our technology [33,46].

## Conclusion

The proposed protocol provides the means for examiners in different locations to remotely administer and score structured motor assessments utilizing a low-cost quantitative continuous measurement of movements in the extremities of people with Parkinson‘s disease [7,8]. The proposed protocol provides the framework for examiners in a spectrum of different locations to remotely view and score structured motor assessments both on videos and on live monitors. The feasibility of remote assessments of patients on videos and on live monitors has been validated by teams of six raters in different locations on three continents [Africa (AE, AOS, HA, MME), Asia (SJ), and North America (JRB)]. The proposed protocol can be utilized by providers, investigators, and patients all in distant locations throughout the world. The proposed protocol will facilitate the remote application of precision medicine to develop treatment plans tailored to the specific needs of individual patients. These protocols can be utilized by colleagues around the world to conduct motor assessments with participants and examiners in vastly different geographical locations. This protocol will provide the bases for telemedicine throughout the world. Utilization of state-of-the-art wearable sensors [10,[34], [35], [36], [37], [38], [39], [40], [41], [42], [43], [44], [45], [46]] will enhance future investigations.

## CRediT authorship contribution statement

**Abdelwahab Elshourbagy:** Conceptualization, Methodology, Software, Validation, Formal analysis, Investigation, Resources, Data curation, Writing – review & editing, Project administration. **Mennatullah Mohamed Eltaras:** Validation, Formal analysis, Investigation, Writing – review & editing. **Hassan Abdalshafy:** Validation, Investigation, Data curation, Writing – review & editing. **Samrah Javed:** Validation, Investigation, Data curation, Writing – review & editing. **Ahmed Omar Sadaney:** Validation, Investigation, Data curation, Writing – review & editing. **Timothy Patrick Harrigan:** Conceptualization, Methodology, Software, Validation, Investigation, Data curation, Writing – review & editing, Project administration, Funding acquisition. **Kelly Alexander Mills:** Investigation, Resources, Data curation, Writing – review & editing, Supervision, Project administration, Funding acquisition. **Manuel Enrique Hernandez:** Conceptualization, Methodology, Software, Writing – review & editing, Supervision, Project administration. **James Robert Brašić:** Conceptualization, Methodology, Software, Validation, Formal analysis, Investigation, Resources, Data curation, Writing – original draft, Writing – review & editing, Visualization, Supervision, Project administration, Funding acquisition.

## Declaration of Competing Interest

The authors declare that they have no known competing financial interests or personal relationships that could have appeared to influence the work reported in this paper.

## Data Availability

All data is provided in the references and the supplementary data.

## Appendix Supplementary materials

Image, application 1

Image, application 2

Image, application 3

Image, application 4

## References

[1] World Medical Association WMA Declaration of Helsinki - Ethical Principles for Medical Research Involving Human Subjects. https://www.wma.net/policies-post/wma-declaration-of-helsinki-ethical-principles-for-medical-research-involving-human-subjects/.

[2] Committee of Medical Journal Editors (ICMJE) (2023). Recommendations for the Conduct, Reporting, Editing, and Publication of Scholarly Work in Medical Journals.

[3] Zirra A., Rao S.C., Bestwick J., Rajalingam R., Marras C., Blauwendraat C., Mata I.F., Noyce A.J. (2022). Gender differences in the prevalence of Parkinson's disease. Mov. Disord. Clin. Pract..

[4] Segura-Aguilar J. (2021).

[5] Zhong Q.Q., Zhu F. (2022). Trends in prevalence cases and disability-adjusted life-years of Parkinson's disease: findings from the Global Burden of Disease Study 2019. Neuroepidemiology.

[6] Goetz C.G., Tilley B.C., Shaftman S.R., Stebbins G.T., Fahn S., Martinez-Martin P. (2008). Movement disorder society-sponsored revision of the unified Parkinson's disease rating scale (MDS-UPDRS)–Scale presentation and clinimetric testing results. Mov. Disord..

[7] McKay G.N., Harrigan T.P., Brasic J.R. (2019). A low-cost quantitative continuous measurement of movements in the extremities of people with Parkinson's disease. MethodsX.

[8] McKay G., Mishra C., Elshourbagy A., Eltaras M., Abdalshafy H., Hernandez M., Brasic J. (2023). Administration of a low-cost quantitative continuous measurement of movements in the extremities of people with Parkinson's disease. Mendeley Data.

[9] Güney G., Jansen T.S., Dill S., Schulz J.B., Dafotakis M., Antin C.H., Braczynski A.K. (2022). Video-based hand movement analysis of Parkinson patients before and after medication using high-frame-rate videos and MediaPipe. Sensors.

[10] Goyal J., Khandnor P., Aseri T.C. (2020). Classification, prediction, and monitoring of Parkinson's disease using computer assisted technologies: a comparative analysis. Eng. Appl. Artif. Intel..

[11] Lu R., Xu Y., Li X., Fan Y., Zeng W., Tan Y., Ren K., Chen W., Cao X. (2020). Evaluation of wearable sensor devices in Parkinson's disease: a review of current status and future prospects. Parkinsons Dis..

[12] Miller-Patterson C., Hsu J.Y., Willis A.W., Hamedani A.G. (2023). Functional impairment in individuals with prodromal or unrecognized Parkinson Disease. JAMA Neurol..

[13] (2023). https://zoom.us.

[14] Bickel P.J., Doksum K.A. (2015).

[15] Harrigan T.P., Hwang B.J., Mathur A.K., Mills K.A., Pantelyat A.Y., Bang J.A. (2020). Dataset of quantitative structured office measurements of movements in the extremities. Data Brief.

[16] Elshourbagy A., Hwang B., Mathur A.K., Mills K., Harrigan T., Brasic J. (2023). A low-cost quantitative continuous measurement of movements in the extremities of healthy men with typical development. Mendeley Data.

[17] Harrigan T., Syed A., Hwang B., Mathur A., Mills K., Pantelyat A., Bang J., Mishra C., Vyas P., Martin S., Jamal A., Ziegelman L., Hernandez M., Shneyderman M., Panaparambil R., Doheim M.F., Gaite A., Wong D., Brasic J. (2022). Quantitative continuous measurement of movements in the extremities. Mendeley Data.

[18] Brasic J.R., Ziegelman L., Kosuri T., Hakim H., Zhao L., Gokce A., Mathur K., Hernandez M.E., Mills K.A. (2022). Classification of extremity movements by visual observation of signal transforms. Mov. Disord..

[19] Kosuri T., Brasic J. (2022). Continuous wavelet transforms to improve the accuracy of motor assessments of Parkinson's disease. Mov. Disord..

[20] Ziegelman L., Kosuri T., Hakim H., Zhao L., Mills K., Hernandez M., Brasic J. (2023). Classification of extremity movements by visual observation of signal transforms. Mendeley Data.

[21] Ziegelman L., Hernandez M., Kosuri T., Martin S., Shneyderman M., Suresh A., Gorny A., Syed A., Qureshi A., Mahmoud A., Dutta A., Jamal A., Bhatnagar A., Udoji C., Cook C., Youssef H., Hakim H., Brookshier I., Kumari K., Tang K., Zhao L., Shankar M., Doheim M., Mansour M., Shehata M., Eltony M., Cho N., Loza N., Thakkar R., Panaparambil R., Bertucci S., Elshourbagy T., Hu Y., Harrigan T., Brasic J. (2022). Signal processing of quantitative continuous measurement of movements in the extremities. Mendeley Data.

[22] Brašić J.R., Ziegelman L., Eltaras M.M., Elshourbagy A., Gupta A., Mills K., Hernandez M.E. (2022). Neurology Exchange Virtual Conference, September 20-22.

[23] Hernandez M.E., Ziegelman L., Kosuri T., Hakim H., Zhao L., Mills K.A., Brašić J.R. (2022). Classification of extremity movements by visual observation of signals and their transforms. MethodsX.

[24] Brasic J.R., Barnett J.Y., Ahn S.C., Nadrich R.H., Will M.V., Clair A. (1997). Clinical assessment of self-injurious behavior. Psychol. Rep..

[25] Brasić J.R., Barnett J.Y., Sheitman B.B., Lafargue R.T., Ahn S.C. (1998). Clinical assessment of adventitious movements. Psychol. Rep..

[26] Elshourbagy A., Hwang B., Mathur A.K., Mills K., Hernandez M., Harrigan T., Pantelyat A., Brasic J. (2023). A low-cost quantitative continuous measurement of movements in the extremities of women with Parkinson's disease. Mendeley Data.

[27] Elshourbagy A., Hwang B., Mathur A.K., Bang J., Mills K., Hernandez M., Harrigan T., Brasic J. (2023). A low-cost quantitative continuous measurement of movements in the extremities of men with Parkinson's disease. Mendeley Data.

[28] Fleiss J.L. (1986).

[29] Elshourbagy A., Eltaras M.M., Abdalshafy H., Sadaney A.O., Harrigan T.P., Mills A., Hernandez M.E., Brasic J.R. (2023). https://www.iaprd-world-congress.com/inhalt/uploads/2023/05/IAPRD2023_Book_of_abstracts.pdf.

[30] Brasic J., Elshourbagy A., Eltaras M., Abdalshafy H., Javed S., Harrigan T., Mills K., Hernandez M. (2023). https://www.iaprd-world-congress.com/inhalt/uploads/2023/05/IAPRD2023_Book_of_abstracts.pdf.

[31] Fleiss J.L., Levin B., Paik M.C. (2003).

[32] Araujo J., Born D.G. (1985). Calculating percentage agreement correctly but writing its formula incorrectly. Behav. Anal..

[33] Suresh A., Hernandez M., Brasic J. (2020). Predicting scores of repetitive movement measurements using image classification. Mendeley Data.

[34] Legaria-Santiago V.K., Sanchez-Fernández L.P., Sanchez-Pérez L.A., Garza-Rodríguez A. (2022). Computer models evaluating hand tremors in Parkinson's disease patients. Comput. Biol. Med..

[35] Dineshkumar V., Dolly D.R.J., Jagannath D.J., Peter J.D. (2022). Assistive methodologies for Parkinson's disease tremor management—a health opinion. Front. Public Health.

[36] Markose N.R., Moyva P.D., Asaithambi M. (2021). Proceedings of 2021 IEEE 7th International Conference on Bio Signals, Images and Instrumentation.

[37] Ingram L.A., Carroll V.K., Butler A.A., Brodie M.A., Gandevia S.C., Lord S.R. (2021). Quantifying upper limb motor impairment in people with Parkinson's disease: a physiological profiling approach. PeerJ.

[38] Rini E.K., Haq E.S. (2021). 2021 International Conference on Computer Science, Information Technology, and Electrical Engineering.

[39] Bobić V., Djurić-Jovičić M., Dragašević N., Popović M.B., Kostić V.S., Kvaščev G. (2019). An expert system for quantification of bradykinesia based on wearable inertial sensors. Sensors (Basel).

[40] Hussain A., Al-Ramadan K. (2022). A core-based XRF scanning workflow for continuous measurement of mineralogical variations in clastic reservoirs. MethodsX.

[41] Foo S.W.L. (2021). Stereoscopic visual stimuli for examining biological motion perception and unanticipated steering manoeuvres in people with Parkinson's disease. MethodsX.

[42] LeMoyne R., Mastroianni T., Grundfest W. (2013). Wireless accelerometer configuration for monitoring Parkinson's disease hand tremor. Adv. Parkinsons Dis..

[43] Liu P., Yu N., Yang Y., Yu Y., Sun X., Yu H., Han J., Wu J. (2022). Quantitative assessment of gait characteristics in patients with Parkinson's disease using 2D video. Parkinson. Relat. Disord..

[44] Liu W., Lin X., Chen X., Wang Q., Wang X., Yang B., Cai N., Chen R., Chen G., Lin Y. (2023). Vision-based estimation of MDS-UPDRS scores for quantifying Parkinson's disease tremor severity. Med. Image Anal..

[45] Lu M., Zhao Q., Poston K.L., Sullivan E.V., Pfefferbaum A., Shahid M., Katz M., Kouhsari L.M., Schulman K., Milstein A., Niebles J.C., Henderson V.W., Fei-Fei L., Pohl K.M., Adel E. (2021). Quantifying Parkinson's disease motor severity under uncertainty using MDS-UPDRS videos. Med. Image Anal..

[46] Prakash P., Kaur R., Levy J., Sowers R., Brašić J., Hernandez M.E. (2023). 45th Annual International Conference of the IEEE Engineering in Medicine and Biology Society (EMBC'23) to be held at the International Convention Centre (ICC) in Sydney Australia.

