# Supplementary material for: Feasibility of virtual low-cost quantitative continuous measurement of movements in the extremities of people with Parkinson’s disease
Source: MethodsX. 2023 Jun 2;11:102230. doi: 10.1016/j.mex.2023.102230 (PMC10293722; doi:10.1016/j.mex.2023.102230)
Supplement: Supplementary file 1 [file mmc1.docx]

Appendix 1

Coding form for a low-cost quantitative continuous measurement of movements

in the extremities of people with Parkinson's disease

Gregory Neal McKay, Timothy P. Harrigan, James Robert Brašić

Patient name _______________________________________________________________________________________

Record number ____________________________________________________________________________________

Date ______________________________________________________________________________________________

Rater _____________________________________________________________________________________________

*● Videotape the participant including the face and the extremities for the entire procedure.*

*● Record on a laptop computer the output of the instrumentation.*

*● Rate live each item immediately after conducting the procedure.*

*● Encircle on the form the behaviors observed and records the laterality (left or right) of the observations.*

*● Verify that the participant is not allergic to hypoallergenic tape.*

*● Fix the accelerometer evaluation boards to the index fingers and wrists as illustrated in Fig. 16 with hypoallergenic tape as follows:*

Accelerometer evaluation board 1 – Dorsal surface of the second (middle) phalanx of the right index finger,

Accelerometer evaluation board 2 – Midway between radius and ulna approximately two inches proximal to the right wrist joint on the dorsum of the arm,

Accelerometer evaluation board 3 – Dorsal surface of the second (middle) phalanx of the left index finger,

Accelerometer evaluation board 4 – Midway between radius and ulna approximately two inches proximal to the left wrist joint on the dorsum of the arm.

“**3.17 Rest tremor amplitude” upper limbs**

“Instructions to examiner: Score the maximum amplitude that is seen at any time as the final score. Rate only the amplitude and not the persistence or the intermittency of the tremor. As part of this rating, the patient should sit quietly in a chair with the hands placed on the arms of the chair (not the lap) and the feet comfortably supported on the floor” for three minutes “with no other directives.” Rest tremor is assessed by viewing both upper limbs jointly during the three minutes. Videotape and record three minutes of resting motion.

“0: Normal: No tremor.

“1: Slight.: < 1 cm in maximal amplitude.

“2: Mild: > 1 cm but < 3 cm in maximal amplitude.

“3: Moderate: 3 - 10 cm in maximal amplitude.

“4: Severe: > 10 cm in maximal amplitude.”

􀀍 􀀍

RUE LUE

**“3.17 Rest tremor amplitude” upper limbs counting**

“Instructions to examiner: Score the maximum amplitude that is seen at any time as the final score. Rate only the amplitude and not the persistence or the intermittency of the tremor. As part of this rating, the patient should sit quietly in a chair with the hands placed on the arms of the chair (not the lap) and the feet comfortably supported on the floor” while counting aloud from 30 backwards. Rest tremor is assessed by viewing both upper limbs jointly. Videotape and record the procedure.

“0: Normal: No tremor.

“1: Slight.: < 1 cm in maximal amplitude.

“2: Mild: > 1 cm but < 3 cm in maximal amplitude.

“3: Moderate: 3 - 10 cm in maximal amplitude.

“4: Severe: > 10 cm in maximal amplitude.”

􀀍 􀀍

RUE LUE

**“3.15 Postural tremor of the hands**

“Instructions to examiner: All tremor, including re-emergent rest tremor, that is present in this posture is to be included in this rating. Rate each hand separately. Rate the highest amplitude seen. Instruct the patient to stretch the arms out in front of the body with palms down. The wrist should be straight, and the fingers comfortably separated so that they do not touch each other. Observe,” videotape, and record *“*this posture for 10 seconds.”

“0: Normal: No tremor.

“1: Slight: Tremor is present but less than 1 cm in amplitude.

“2: Mild: Tremor is at least 1 but less than 3 cm in amplitude.

“3: Moderate: Tremor is at least 3 but less than 10 cm in amplitude.

“4: Severe: Tremor is at least 10 cm in amplitude.”

􀀍 􀀍

RUE LUE

**“3.4 Finger tapping**

“Instructions to examiner: Each hand is tested separately. Demonstrate the task, but do not continue to perform the task while the patient is being tested. Instruct the patient to tap the index finger on the thumb 10 times as quickly AND as big as possible. Rate each side separately, evaluating speed, amplitude, hesitations, halts, and decrementing amplitude.

“0: Normal: No problems.

“1: Slight: Any of the following: a) the regular rhythm is broken with one or two interruptions or hesitations of the tapping movement; b) slight slowing; c) the amplitude decrements near the end of the 10 taps.

“2: Mild: Any of the following: a) 3 to 5 interruptions during tapping; b) mild slowing; c) the amplitude decrements midway in the 10-tap sequence.

“3: Moderate: Any of the following: a) more than 5 interruptions during tapping or at least one long arrest (freeze) in ongoing movement; b) moderate slowing; c) the amplitude decrements starting after the 1st tap.

“4: Severe: Cannot or can only barely perform the task because of slowing, interruptions or decrements.”

􀀍 􀀍

RUE LUE

**“3.5 Hand movements**

“Instructions to examiner: Test each hand separately. Demonstrate the task, but do not continue to perform the task while the patient is being tested. Instruct the patient to make a tight fist with the arm bent at the elbow so that the palm faces the examiner. Have the patient open the hand 10 times as fully AND as quickly as possible. If the patient fails to make a tight fist or to open the hand fully, remind him/her to do so. Rate each side separately, evaluating speed, amplitude, hesitations, halts, and decrementing amplitude.

“0: Normal: No problem.

“1: Slight: Any of the following: a) the regular rhythm is broken with one or two interruptions or hesitations of the movement; b) slight slowing; c) the amplitude decrements near the end of the task.

“2: Mild: Any of the following: a) 3 to 5 interruptions during the movements; b) mild slowing; c) the amplitude decrements midway in the task.

“3: Moderate: Any of the following: a) more than 5 interruptions during the movement or at least one long arrest (freeze) in ongoing movement; b) moderate slowing; c) the amplitude decrements starting after the 1st open-and-close sequence.

“4: Severe: Cannot or can only barely perform the task because of slowing, interruptions or decrements.

􀀍 􀀍

RUE LUE

**“3.6 Pronation-supination movements of the hands**

“Instructions to examiner: Test each hand separately. Demonstrate the task, but do not continue to perform the task while the patient is being tested. Instruct the patient to extend the arm out in front of his/her body with the palms down; then to turn the palm up and down alternately 10 times as fast and as fully as possible. Rate each side separately, evaluating speed, amplitude, hesitations, halts, and decrementing amplitude.

“0: Normal: No problems.

“1: Slight: Any of the following: a) the regular rhythm is broken with one or two interruptions or hesitations of the movement; b) slight slowing; c) the amplitude decrements near the end of the sequence.

“2: Mild: Any of the following: a) 3 to 5 interruptions during the movements; b) mild slowing; c) the amplitude decrements midway in the sequence.

“3: Moderate: Any of the following: a) more than 5 interruptions during the movement or at least one long arrest (freeze) in ongoing movement; b) moderate slowing c) the amplitude decrements starting after the 1st supination-pronation sequence.

“4: Severe: Cannot or can only barely perform the task because of slowing, interruptions or decrements.

􀀍 􀀍

RUE LUE

**“3.9 Arising from chair” upper limbs**

“Instructions to examiner: Have the patient sit in a straight-backed chair with arms, with both feet on the floor and sitting back in the chair (if the patient is not too short). Ask the patient to cross his/her arms across the chest and then to stand up. If the patient is not successful, repeat this attempt a maximum up to two more times. If still unsuccessful, allow the patient to move forward in the chair to arise with arms folded across the chest. Allow only one attempt in this situation. If unsuccessful, allow the patient to push off using his/her hands on the arms of the chair. Allow a maximum of three trials of pushing off. If still not successful, assist the patient to arise.

“0: Normal: No problems. Able to arise quickly without hesitation.

“1: Slight: Arising is slower than normal; or may need more than one attempt; or may need to move forward in the chair to arise. No need to use the arms of the chair.

“2: Mild: Pushes self up from arms of chair without difficulty.

“3: Moderate: Needs to push off, but tends to fall back; or may have to try more than one time using arms of chair, but can get up without help.

“4: Severe: Unable to arise without help.

􀀍

*●Remove the accelerometer evaluation boards from the fingers and wrists.*

*● Fix the accelerometer evaluation boards to the shins and big toes as illustrated in Figure 17 with hypoallergenic tape as follows:*

Accelerometer evaluation board 1 – Anterior surface of the right tibia two inches proximal to the medial malleolus.

Accelerometer evaluation board 2 – Dorsal surface of the proximal phalanx of the right big toe.

Accelerometer evaluation board 3 – Anterior surface of the left tibia two inches proximal to the medial malleolus.

Accelerometer evaluation board 4 – Dorsal surface of the proximal phalanx of the left big toe.

**“3.9 Arising from chair” lower limbs**

“Instructions to examiner: Have the patient sit in a straight-backed chair with arms, with both feet on the floor and sitting back in the chair (if the patient is not too short). Ask the patient to cross his/her arms across the chest and then to stand up. If the patient is not successful, repeat this attempt a maximum up to two more times. If still unsuccessful, allow the patient to move forward in the chair to arise with arms folded across the chest. Allow only one attempt in this situation. If unsuccessful, allow the patient to push off using his/her hands on the arms of the chair. Allow a maximum of three trials of pushing off. If still not successful, assist the patient to arise.

“0: Normal: No problems. Able to arise quickly without hesitation.

“1: Slight: Arising is slower than normal; or may need more than one attempt; or may need to move forward in the chair to arise. No need to use the arms of the chair.

“2: Mild: Pushes self up from arms of chair without difficulty.

“3: Moderate: Needs to push off, but tends to fall back; or may have to try more than one time using arms of chair, but can get up without help.

“4: Severe: Unable to arise without help.

􀀍

“**3.17 Rest tremor amplitude” lower limbs**

“Instructions to examiner: Score the maximum amplitude that is seen at any time as the final score. Rate only the amplitude and not the persistence or the intermittency of the tremor. As part of this rating, the patient should sit quietly in a chair with the hands placed on the arms of the chair (not the lap) and the feet comfortably supported on the floor” for three minutes “with no other directives.” Rest tremor is assessed by viewing both lower limbs jointly during the three minutes. Videotape and record three minutes of resting motion.

“Extremity ratings

“0: Normal: No tremor.

“1: Slight: < 1 cm in maximal amplitude.

“2: Mild: > 1 cm but < 3 cm in maximal amplitude.

“3: Moderate: 3 - 10 cm in maximal amplitude.

“4: Severe: > 10 cm in maximal amplitude.”

􀀍 􀀍

RLE LLE

**“3.17 Rest tremor amplitude” lower limbs counting**

“Instructions to examiner: Score the maximum amplitude that is seen at any time as the final score. Rate only the amplitude and not the persistence or the intermittency of the tremor. As part of this rating, the patient should sit quietly in a chair with the hands placed on the arms of the chair (not the lap) and the feet comfortably supported on the floor”

while counting aloud from 30 backwards. Rest tremor is assessed by viewing both lower limbs jointly. Videotape and record the procedure.

“Extremity ratings

“0: Normal: No tremor.

“1: Slight: < 1 cm in maximal amplitude.

“2: Mild: > 1 cm but < 3 cm in maximal amplitude.

“3: Moderate: 3 - 10 cm in maximal amplitude.

“4: Severe: > 10 cm in maximal amplitude.”

􀀍 􀀍

RLE LLE

**“3.7 Toe tapping**

“Instructions to examiner: Have the patient sit in a straight-backed chair with arms, both feet on the floor. Test each foot separately. Demonstrate the task, but do not continue to perform the task while the patient is being tested. Instruct the patient to place the heel on the ground in a comfortable position and then tap the toes 10 times as big and as fast as possible. Rate each side separately, evaluating speed, amplitude, hesitations, halts, and decrementing amplitude.

“0: Normal: No problem.

“1: Slight: Any of the following: a) the regular rhythm is broken with one or two interruptions or hesitations of the tapping movement; b) slight slowing; c) amplitude decrements near the end of the ten taps.

“2: Mild: Any of the following: a) 3 to 5 interruptions during the tapping movements; b) mild slowing; c) amplitude decrements midway in the task.

“3: Moderate: Any of the following: a) more than 5 interruptions during the tapping movements or at least one long arrest (freeze) in ongoing movement; b) moderate slowing; c) amplitude decrements after the first tap.

“4: Severe: Cannot or can only barely perform the task because of slowing, interruptions or decrements.

􀀍 􀀍

RLE LLE

**“3.8 Leg agility**

“Instructions to examiner: Have the patient sit in a straight-backed chair with arms. The patient should have both feet comfortably on the floor. Test each leg separately. Demonstrate the task, but do not continue to perform the task while the patient is being tested. Instruct the patient to place the foot on the ground in a comfortable position and then raise and stomp the foot on the ground 10 times as high and as fast as possible. Rate each side separately, evaluating speed, amplitude, hesitations, halts, and decrementing amplitude.

“0: Normal: No problems.

“1: Slight: Any of the following: a) the regular rhythm is broken with one or two interruptions or hesitations of the movement; b) slight slowing; c) amplitude decrements near the end of the task.

“2: Mild: Any of the following: a) 3 to 5 interruptions during the movements; b) mild slowing; c) amplitude decrements midway in the task.

“3: Moderate: Any of the following: a) more than 5 interruptions during the movement or at least one long arrest (freeze) in ongoing movement; b) moderate slowing in speed; c) amplitude decrements after the first tap.

“4: Severe: Cannot or can only barely perform the task because of slowing, interruptions or decrements.

􀀍 􀀍

RLE LLE

Portions of the Movement Disorder Society-Sponsored Revision of the Unified Parkinson’s Disease Rating Scale (MDS-UPDRS) (Goetz et al., 2008) in quotation marks are used with the kind permission of the International Parkinson and Movement Disorder Society.

Reproduced with permission [7].
