# Supplementary material for: Feasibility of virtual low-cost quantitative continuous measurement of movements in the extremities of people with Parkinson’s disease
Source: MethodsX. 2023 Jun 2;11:102230. doi: 10.1016/j.mex.2023.102230 (PMC10293722; doi:10.1016/j.mex.2023.102230)
Supplement: Supplementary file 2 [file mmc2.pdf]

Marketplace

This is a License Agreement between James Robert Brasic ("User") and Copyright Clearance Center, Inc. ("CCC") on behalf of the Rightsholder identified in the order details below. The license consists of the order details, the Marketplace Permissions General Terms and Conditions below, and any Rightsholder Terms and Conditions which are included below.

All payments must be made in full to CCC in accordance with the Marketplace Permissions General Terms and Conditions below.

**Order Date**	01-Apr-2023
**Order License ID**	1340582-1
**ISSN**	2215-0161
**Type of Use**	Republish in a journal/magazine
**Publisher**	Elsevier
**Portion**	Chart/graph/table/figure

LICENSED CONTENT

**Publication Title**	MethodsX
**Article Title**	A low-cost quantitative continuous measurement of movements in the extremities of people with Parkinson's disease.
**Date**	01/01/2014
**Language**	English
**Country**	United Kingdom of Great Britain and Northern Ireland
**Rightsholder**	Elsevier Science & Technology Journals
**Publication Type**	e-Journal
**Start Page**	169
**End Page**	189
**Volume**	6

REQUEST DETAILS

**Portion Type**	Chart/graph/table/figure
**Number of Charts / Graphs / Tables / Figures Requested**	1
**Format (select all that apply)**	Print, Electronic
**Who Will Republish the Content?**	Author of requested content
**Duration of Use**	Life of current edition
**Lifetime Unit Quantity**	Up to 44,999
**Rights Requested**	Main product, any product related to main product, and other compilations/derivative products
**Distribution**	Worldwide
**Translation**	Original language of publication
**Copies for the Disabled?**	Yes
**Minor Editing Privileges?**	Yes
**Incidental Promotional Use?**	Yes
**Currency**	USD

NEW WORK DETAILS

**Title**	Virtual low-cost quantitative continuous measurement of movements in the extremities of people with Parkinson's disease	**Publisher Imprint**	Elsevier
**Author**	Abdelwahab Elshourbagy, Mennatullah Mohamed Eltaras, Hassan Abdalshafy, Samrah Javed, Ahmed O. Sadaney, Timothy P. Harrigan, Kelly Alexander Mills, Manuel Enrique Hernandez, James Robert Brašić	**Expected Publication Date**	2023-05-01
**Publication**	MethodsX	**Expected Size of the New Work (number of pages)**	30
**Publisher**	Elsevier	**Standard Identifier**	Virtual low-cost quantitative continuous

ADDITIONAL DETAILS

**Order Reference Number**	N/A	**The Requesting Person/Organization to Appear on the License**	James Robert Brasic

REQUESTED CONTENT DETAILS

**Title, Description or Numeric Reference of the Portion(s)**	Appendix	**Title of the Article/Chapter the Portion Is From**	A low-cost quantitative continuous measurement of movements in the extremities of people with Parkinson's disease.
**Editor of Portion(s)**	McKay, Gregory Neal; Harrigan, Timothy P.; Bra?i?, James Robert	**Author of Portion(s)**	McKay, Gregory Neal; Harrigan, Timothy P.; Bra?i?, James Robert
**Volume of Serial or Monograph**	6	**Issue, if Republishing an Article From a Serial**	N/A
**Page or Page Range of Portion**	169-189	**Publication Date of Portion**	2019-01-01

RIGHTSHOLDER TERMS AND CONDITIONS

Elsevier publishes Open Access articles in both its Open Access journals and via its Open Access articles option in subscription journals, for which an author selects a user license permitting certain types of reuse without permission. Before proceeding please check if the article is Open Access on http://www.sciencedirect.com and refer to the user license for the individual article. Any reuse not included in the user license terms will require permission. You must always fully and appropriately credit the author and source. If any part of the material to be used (for example, figures) has appeared in the Elsevier publication for which you are seeking permission, with credit or acknowledgement to another source it is the responsibility of the user to ensure their reuse complies with the terms and conditions determined by the rights holder. Please contact permissions@elsevier.com with any queries.

Marketplace Permissions General Terms and Conditions

The following terms and conditions ("General Terms"), together with any applicable Publisher Terms and Conditions, govern User's use of Works pursuant to the Licenses granted by Copyright Clearance Center, Inc. ("CCC") on behalf of the

applicable Rightsholders of such Works through CCC's applicable Marketplace transactional licensing services (each, a "Service").

1) **Definitions.** For purposes of these General Terms, the following definitions apply:

"License" is the licensed use the User obtains via the Marketplace platform in a particular licensing transaction, as set forth in the Order Confirmation.

"Order Confirmation" is the confirmation CCC provides to the User at the conclusion of each Marketplace transaction. "Order Confirmation Terms" are additional terms set forth on specific Order Confirmations not set forth in the General Terms that can include terms applicable to a particular CCC transactional licensing service and/or any Rightsholder-specific terms.

"Rightsholder(s)" are the holders of copyright rights in the Works for which a User obtains licenses via the Marketplace platform, which are displayed on specific Order Confirmations.

"Terms" means the terms and conditions set forth in these General Terms and any additional Order Confirmation Terms collectively.

"User" or "you" is the person or entity making the use granted under the relevant License. Where the person accepting the Terms on behalf of a User is a freelancer or other third party who the User authorized to accept the General Terms on the User's behalf, such person shall be deemed jointly a User for purposes of such Terms.

"Work(s)" are the copyright protected works described in relevant Order Confirmations.

2) **Description of Service.** CCC's Marketplace enables Users to obtain Licenses to use one or more Works in accordance with all relevant Terms. CCC grants Licenses as an agent on behalf of the copyright rightsholder identified in the relevant Order Confirmation.

3) **Applicability of Terms.** The Terms govern User's use of Works in connection with the relevant License. In the event of any conflict between General Terms and Order Confirmation Terms, the latter shall govern. User acknowledges that Rightsholders have complete discretion whether to grant any permission, and whether to place any limitations on any grant, and that CCC has no right to supersede or to modify any such discretionary act by a Rightsholder.

4) **Representations; Acceptance.** By using the Service, User represents and warrants that User has been duly authorized by the User to accept, and hereby does accept, all Terms.

5) **Scope of License; Limitations and Obligations.** All Works and all rights therein, including copyright rights, remain the sole and exclusive property of the Rightsholder. The License provides only those rights expressly set forth in the terms and conveys no other rights in any Works

6) **General Payment Terms.** User may pay at time of checkout by credit card or choose to be invoiced. If the User chooses to be invoiced, the User shall: (i) remit payments in the manner identified on specific invoices, (ii) unless otherwise specifically stated in an Order Confirmation or separate written agreement, Users shall remit payments upon receipt of the relevant invoice from CCC, either by delivery or notification of availability of the invoice via the Marketplace platform, and (iii) if the User does not pay the invoice within 30 days of receipt, the User may incur a service charge of 1.5% per month or the maximum rate allowed by applicable law, whichever is less. While User may exercise the rights in the License immediately upon receiving the Order Confirmation, the License is automatically revoked and is null and void, as if it had never been issued, if CCC does not receive complete payment on a timely basis.

7) **General Limits on Use.** Unless otherwise provided in the Order Confirmation, any grant of rights to User (i) involves only the rights set forth in the Terms and does not include subsequent or additional uses, (ii) is non-exclusive and non-transferable, and (iii) is subject to any and all limitations and restrictions (such as, but not limited to, limitations on duration of use or circulation) included in the Terms. Upon completion of the licensed use as set forth in the Order Confirmation, User shall either secure a new permission for further use of the Work(s) or immediately cease any new use of the Work(s) and shall render inaccessible (such as by deleting or by removing or severing links or other locators) any further copies of the Work. User may only make alterations to the Work if and as expressly set forth in the Order Confirmation. No Work may be used in any way that is unlawful, including without limitation if such use would violate applicable sanctions laws or regulations, would be defamatory, violate the rights of third parties (including such third parties' rights of copyright, privacy, publicity, or other tangible or intangible property), or is otherwise illegal, sexually explicit, or obscene. In addition, User may not conjoin a Work with any other material that may result in damage to the

reputation of the Rightsholder. Any unlawful use will render any licenses hereunder null and void. User agrees to inform CCC if it becomes aware of any infringement of any rights in a Work and to cooperate with any reasonable request of CCC or the Rightsholder in connection therewith.

8) **Third Party Materials.** In the event that the material for which a License is sought includes third party materials (such as photographs, illustrations, graphs, inserts and similar materials) that are identified in such material as having been used by permission (or a similar indicator), User is responsible for identifying, and seeking separate licenses (under this Service, if available, or otherwise) for any of such third party materials; without a separate license, User may not use such third party materials via the License.

9) **Copyright Notice.** Use of proper copyright notice for a Work is required as a condition of any License granted under the Service. Unless otherwise provided in the Order Confirmation, a proper copyright notice will read substantially as follows: "Used with permission of [Rightsholder's name], from [Work's title, author, volume, edition number and year of copyright]; permission conveyed through Copyright Clearance Center, Inc." Such notice must be provided in a reasonably legible font size and must be placed either on a cover page or in another location that any person, upon gaining access to the material which is the subject of a permission, shall see, or in the case of republication Licenses, immediately adjacent to the Work as used (for example, as part of a by-line or footnote) or in the place where substantially all other credits or notices for the new work containing the republished Work are located. Failure to include the required notice results in loss to the Rightsholder and CCC, and the User shall be liable to pay liquidated damages for each such failure equal to twice the use fee specified in the Order Confirmation, in addition to the use fee itself and any other fees and charges specified.

10) **Indemnity.** User hereby indemnifies and agrees to defend the Rightsholder and CCC, and their respective employees and directors, against all claims, liability, damages, costs, and expenses, including legal fees and expenses, arising out of any use of a Work beyond the scope of the rights granted herein and in the Order Confirmation, or any use of a Work which has been altered in any unauthorized way by User, including claims of defamation or infringement of rights of copyright, publicity, privacy, or other tangible or intangible property.

11) **Limitation of Liability.** UNDER NO CIRCUMSTANCES WILL CCC OR THE RIGHTSHOLDER BE LIABLE FOR ANY DIRECT, INDIRECT, CONSEQUENTIAL, OR INCIDENTAL DAMAGES (INCLUDING WITHOUT LIMITATION DAMAGES FOR LOSS OF BUSINESS PROFITS OR INFORMATION, OR FOR BUSINESS INTERRUPTION) ARISING OUT OF THE USE OR INABILITY TO USE A WORK, EVEN IF ONE OR BOTH OF THEM HAS BEEN ADVISED OF THE POSSIBILITY OF SUCH DAMAGES. In any event, the total liability of the Rightsholder and CCC (including their respective employees and directors) shall not exceed the total amount actually paid by User for the relevant License. User assumes full liability for the actions and omissions of its principals, employees, agents, affiliates, successors, and assigns.

12) **Limited Warranties.** THE WORK(S) AND RIGHT(S) ARE PROVIDED "AS IS." CCC HAS THE RIGHT TO GRANT TO USER THE RIGHTS GRANTED IN THE ORDER CONFIRMATION DOCUMENT. CCC AND THE RIGHTSHOLDER DISCLAIM ALL OTHER WARRANTIES RELATING TO THE WORK(S) AND RIGHT(S), EITHER EXPRESS OR IMPLIED, INCLUDING WITHOUT LIMITATION IMPLIED WARRANTIES OF MERCHANTABILITY OR FITNESS FOR A PARTICULAR PURPOSE. ADDITIONAL RIGHTS MAY BE REQUIRED TO USE ILLUSTRATIONS, GRAPHS, PHOTOGRAPHS, ABSTRACTS, INSERTS, OR OTHER PORTIONS OF THE WORK (AS OPPOSED TO THE ENTIRE WORK) IN A MANNER CONTEMPLATED BY USER; USER UNDERSTANDS AND AGREES THAT NEITHER CCC NOR THE RIGHTSHOLDER MAY HAVE SUCH ADDITIONAL RIGHTS TO GRANT.

13) **Effect of Breach.** Any failure by User to pay any amount when due, or any use by User of a Work beyond the scope of the License set forth in the Order Confirmation and/or the Terms, shall be a material breach of such License. Any breach not cured within 10 days of written notice thereof shall result in immediate termination of such License without further notice. Any unauthorized (but licensable) use of a Work that is terminated immediately upon notice thereof may be liquidated by payment of the Rightsholder's ordinary license price therefor; any unauthorized (and unlicensable) use that is not terminated immediately for any reason (including, for example, because materials containing the Work cannot reasonably be recalled) will be subject to all remedies available at law or in equity, but in no event to a payment of less than three times the Rightsholder's ordinary license price for the most closely analogous licensable use plus Rightsholder's and/or CCC's costs and expenses incurred in collecting such payment.

14) **Additional Terms for Specific Products and Services.** If a User is making one of the uses described in this Section 14, the additional terms and conditions apply:

a) ***Print Uses of Academic Course Content and Materials (photocopies for academic coursepacks or classroom handouts).*** For photocopies for academic coursepacks or classroom handouts the following additional terms apply:

i) The copies and anthologies created under this License may be made and assembled by faculty members individually or at their request by on-campus bookstores or copy centers, or by off-campus copy shops and other similar entities.

ii) No License granted shall in any way: (i) include any right by User to create a substantively non-identical copy of the Work or to edit or in any other way modify the Work (except by means of deleting material immediately preceding or following the entire portion of the Work copied) (ii) permit "publishing ventures" where any particular anthology would be systematically marketed at multiple institutions.

iii) Subject to any Publisher Terms (and notwithstanding any apparent contradiction in the Order Confirmation arising from data provided by User), any use authorized under the academic pay-per-use service is limited as follows:

A) any License granted shall apply to only one class (bearing a unique identifier as assigned by the institution, and thereby including all sections or other subparts of the class) at one institution;

B) use is limited to not more than 25% of the text of a book or of the items in a published collection of essays, poems or articles;

C) use is limited to no more than the greater of (a) 25% of the text of an issue of a journal or other periodical or (b) two articles from such an issue;

D) no User may sell or distribute any particular anthology, whether photocopied or electronic, at more than one institution of learning;

E) in the case of a photocopy permission, no materials may be entered into electronic memory by User except in order to produce an identical copy of a Work before or during the academic term (or analogous period) as to which any particular permission is granted. In the event that User shall choose to retain materials that are the subject of a photocopy permission in electronic memory for purposes of producing identical copies more than one day after such retention (but still within the scope of any permission granted), User must notify CCC of such fact in the applicable permission request and such retention shall constitute one copy actually sold for purposes of calculating permission fees due; and

F) any permission granted shall expire at the end of the class. No permission granted shall in any way include any right by User to create a substantively non-identical copy of the Work or to edit or in any other way modify the Work (except by means of deleting material immediately preceding or following the entire portion of the Work copied).

iv) Books and Records; Right to Audit. As to each permission granted under the academic pay-per-use Service, User shall maintain for at least four full calendar years books and records sufficient for CCC to determine the numbers of copies made by User under such permission. CCC and any representatives it may designate shall have the right to audit such books and records at any time during User's ordinary business hours, upon two days' prior notice. If any such audit shall determine that User shall have underpaid for, or underreported, any photocopies sold or by three percent (3%) or more, then User shall bear all the costs of any such audit; otherwise, CCC shall bear the costs of any such audit. Any amount determined by such audit to have been underpaid by User shall immediately be paid to CCC by User, together with interest thereon at the rate of 10% per annum from the date such amount was originally due. The provisions of this paragraph shall survive the termination of this License for any reason.

b) ***Digital Pay-Per-Uses of Academic Course Content and Materials (e-coursepacks, electronic reserves, learning management systems, academic institution intranets).*** For uses in e-coursepacks, posts in electronic reserves, posts in learning management systems, or posts on academic institution intranets, the following additional terms apply:

i) The pay-per-uses subject to this Section 14(b) include:

A) **Posting e-reserves, course management systems, e-coursepacks for text-based content,** which grants authorizations to import requested material in electronic format, and allows electronic access to this material to members of a designated college or university class, under the direction of an instructor designated by the college or university, accessible only under appropriate electronic controls (e.g., password);

B) **Posting e-reserves, course management systems, e-coursepacks for material consisting of photographs or other still images not embedded in text,** which grants not only the authorizations described in Section 14(b)(i)(A) above, but also the following authorization: to include the requested material in course materials for use consistent with Section 14(b)(i)(A) above, including any necessary resizing, reformatting or modification of the resolution of such requested material (provided that such modification does not alter the underlying editorial content or meaning of the requested material, and provided that the resulting modified content is used solely within the scope of, and in a manner consistent with, the particular authorization described in the Order Confirmation and the Terms), but not including any other form of manipulation, alteration or editing of the requested material;

C) **Posting e-reserves, course management systems, e-coursepacks or other academic distribution for audiovisual content,** which grants not only the authorizations described in Section 14(b)(i)(A) above, but also the following authorizations: (i) to include the requested material in course materials for use consistent with Section 14(b)(i)(A) above; (ii) to display and perform the requested material to such members of such class in the physical classroom or remotely by means of streaming media or other video formats; and (iii) to "clip" or reformat the requested material for purposes of time or content management or ease of delivery, provided that such “clipping” or reformatting does not alter the underlying editorial content or meaning of the requested material and that the resulting material is used solely within the scope of, and in a manner consistent with, the particular authorization described in the Order Confirmation and the Terms. Unless expressly set forth in the relevant Order Conformation, the License does not authorize any other form of manipulation, alteration or editing of the requested material.

ii) Unless expressly set forth in the relevant Order Confirmation, no License granted shall in any way: (i) include any right by User to create a substantively non-identical copy of the Work or to edit or in any other way modify the Work (except by means of deleting material immediately preceding or following the entire portion of the Work copied or, in the case of Works subject to Sections 14(b)(1)(B) or (C) above, as described in such Sections) (ii) permit "publishing ventures" where any particular course materials would be systematically marketed at multiple institutions.

iii) Subject to any further limitations determined in the Rightsholder Terms (and notwithstanding any apparent contradiction in the Order Confirmation arising from data provided by User), any use authorized under the electronic course content pay-per-use service is limited as follows:

A) any License granted shall apply to only one class (bearing a unique identifier as assigned by the institution, and thereby including all sections or other subparts of the class) at one institution;

B) use is limited to not more than 25% of the text of a book or of the items in a published collection of essays, poems or articles;

C) use is limited to not more than the greater of (a) 25% of the text of an issue of a journal or other periodical or (b) two articles from such an issue;

D) no User may sell or distribute any particular materials, whether photocopied or electronic, at more than one institution of learning;

E) electronic access to material which is the subject of an electronic-use permission must be limited by means of electronic password, student identification or other control permitting access solely to students and instructors in the class;

F) User must ensure (through use of an electronic cover page or other appropriate means) that any person, upon gaining electronic access to the material, which is the subject of a permission, shall see:

- a proper copyright notice, identifying the Rightsholder in whose name CCC has granted permission,
- a statement to the effect that such copy was made pursuant to permission,
- a statement identifying the class to which the material applies and notifying the reader that the material has been made available electronically solely for use in the class, and
- a statement to the effect that the material may not be further distributed to any person outside the class, whether by copying or by transmission and whether electronically or in paper form, and User must also

ensure that such cover page or other means will print out in the event that the person accessing the material chooses to print out the material or any part thereof.

G) any permission granted shall expire at the end of the class and, absent some other form of authorization, User is thereupon required to delete the applicable material from any electronic storage or to block electronic access to the applicable material.

iv) Uses of separate portions of a Work, even if they are to be included in the same course material or the same university or college class, require separate permissions under the electronic course content pay-per-use Service. Unless otherwise provided in the Order Confirmation, any grant of rights to User is limited to use completed no later than the end of the academic term (or analogous period) as to which any particular permission is granted.

v) Books and Records; Right to Audit. As to each permission granted under the electronic course content Service, User shall maintain for at least four full calendar years books and records sufficient for CCC to determine the numbers of copies made by User under such permission. CCC and any representatives it may designate shall have the right to audit such books and records at any time during User's ordinary business hours, upon two days' prior notice. If any such audit shall determine that User shall have underpaid for, or underreported, any electronic copies used by three percent (3%) or more, then User shall bear all the costs of any such audit; otherwise, CCC shall bear the costs of any such audit. Any amount determined by such audit to have been underpaid by User shall immediately be paid to CCC by User, together with interest thereon at the rate of 10% per annum from the date such amount was originally due. The provisions of this paragraph shall survive the termination of this license for any reason.

c) ***Pay-Per-Use Permissions for Certain Reproductions (Academic photocopies for library reserves and interlibrary loan reporting) (Non-academic internal/external business uses and commercial document delivery).*** The License expressly excludes the uses listed in Section (c)(i)-(v) below (which must be subject to separate license from the applicable Rightsholder) for: academic photocopies for library reserves and interlibrary loan reporting; and non-academic internal/external business uses and commercial document delivery.

i) electronic storage of any reproduction (whether in plain-text, PDF, or any other format) other than on a transitory basis;

ii) the input of Works or reproductions thereof into any computerized database;

iii) reproduction of an entire Work (cover-to-cover copying) except where the Work is a single article;

iv) reproduction for resale to anyone other than a specific customer of User;

v) republication in any different form. Please obtain authorizations for these uses through other CCC services or directly from the rightsholder.

Any license granted is further limited as set forth in any restrictions included in the Order Confirmation and/or in these Terms.

d) ***Electronic Reproductions in Online Environments (Non-Academic-email, intranet, internet and extranet).*** For "electronic reproductions", which generally includes e-mail use (including instant messaging or other electronic transmission to a defined group of recipients) or posting on an intranet, extranet or Intranet site (including any display or performance incidental thereto), the following additional terms apply:

i) Unless otherwise set forth in the Order Confirmation, the License is limited to use completed within 30 days for any use on the Internet, 60 days for any use on an intranet or extranet and one year for any other use, all as measured from the "republication date" as identified in the Order Confirmation, if any, and otherwise from the date of the Order Confirmation.

ii) User may not make or permit any alterations to the Work, unless expressly set forth in the Order Confirmation (after request by User and approval by Rightsholder); provided, however, that a Work consisting of photographs or other still images not embedded in text may, if necessary, be resized, reformatted or have its resolution modified without additional express permission, and a Work consisting of audiovisual content may, if necessary, be "clipped" or reformatted for purposes of time or content management or ease of delivery (provided that any such resizing, reformatting, resolution modification or “clipping” does not alter the underlying editorial content or

meaning of the Work used, and that the resulting material is used solely within the scope of, and in a manner consistent with, the particular License described in the Order Confirmation and the Terms.

15) **Miscellaneous.**

a) User acknowledges that CCC may, from time to time, make changes or additions to the Service or to the Terms, and that Rightsholder may make changes or additions to the Rightsholder Terms. Such updated Terms will replace the prior terms and conditions in the order workflow and shall be effective as to any subsequent Licenses but shall not apply to Licenses already granted and paid for under a prior set of terms.

b) Use of User-related information collected through the Service is governed by CCC's privacy policy, available online at www.copyright.com/about/privacy-policy/.

c) The License is personal to User. Therefore, User may not assign or transfer to any other person (whether a natural person or an organization of any kind) the License or any rights granted thereunder; provided, however, that, where applicable, User may assign such License in its entirety on written notice to CCC in the event of a transfer of all or substantially all of User's rights in any new material which includes the Work(s) licensed under this Service.

d) No amendment or waiver of any Terms is binding unless set forth in writing and signed by the appropriate parties, including, where applicable, the Rightsholder. The Rightsholder and CCC hereby object to any terms contained in any writing prepared by or on behalf of the User or its principals, employees, agents or affiliates and purporting to govern or otherwise relate to the License described in the Order Confirmation, which terms are in any way inconsistent with any Terms set forth in the Order Confirmation, and/or in CCC's standard operating procedures, whether such writing is prepared prior to, simultaneously with or subsequent to the Order Confirmation, and whether such writing appears on a copy of the Order Confirmation or in a separate instrument.

e) The License described in the Order Confirmation shall be governed by and construed under the law of the State of New York, USA, without regard to the principles thereof of conflicts of law. Any case, controversy, suit, action, or proceeding arising out of, in connection with, or related to such License shall be brought, at CCC's sole discretion, in any federal or state court located in the County of New York, State of New York, USA, or in any federal or state court whose geographical jurisdiction covers the location of the Rightsholder set forth in the Order Confirmation. The parties expressly submit to the personal jurisdiction and venue of each such federal or state court.

Last updated October 2022
