# Supplementary material for: Feasibility of virtual low-cost quantitative continuous measurement of movements in the extremities of people with Parkinson’s disease
Source: MethodsX. 2023 Jun 2;11:102230. doi: 10.1016/j.mex.2023.102230 (PMC10293722; doi:10.1016/j.mex.2023.102230)
Supplement: Supplementary file 4 [file mmc4.docx]

Supplementary Table 1. Independent scores (0,1,2,3,4) of six independent raters of videos of the motor tasks of a low-cost quantitative continuous measurement of movements in the extremities of people with Parkinson's disease [7] of five participants and the corresponding percentage agreements for consensus scores represented by %. The headings include the number and abbreviation of each task (See Appendix 1 [7] in Supplementary materials) and the initials of the individual raters or the consensus score represented by % for percentage agreement.

.

.

.

.

.

.

0

.

.

Age: Age in years; SD: Standard deviation; 3.17 RTUR: 3.17 Rest tremor amplitude upper limbs right; 3.17 RTUL: 3.17 Rest tremor amplitude upper limbs left; 3.17 RTUCR: 3.17 Rest tremor amplitude upper limbs counting right; 3.17 RTUCL: 3.17 Rest tremor amplitude upper limbs counting left; 3.15 PTR: 3.15 Postural tremor of the hands right; 3.15 PTL: 3.15 Postural tremor of the hands left; 3.4 FTR: 3.4 Finger tapping right; 3.4 FTL: 3.4 Finger tapping left; 3.5 HMR: 3.5 Hand movements right; 3.5 HML: 3.5 Hand movements left; 3.6PSR: 3.6 Pronation-supination movements of the hands right; 3.6 PSL: 3.6 Pronation-supination movements of the hands left; 3.9 ACU: 3.9 Arising from chair upper limbs; 3.17 RTLR: 3.17 Rest tremor amplitude lower limbs right; 3.17 RTLL: 3.17 Rest tremor amplitude lower limbs left; 3.17 RTLCR: 3.17 Rest tremor amplitude lower limbs counting right; 3.17 RTLCL: 3.17 Rest tremor amplitude lower limbs counting left; 3.7 TTR: 3.7 Toe tapping right; 3.7 TTL: 3.7 Toe tapping left; 3.8 LAR: 3.8 Leg agility right; 3.8 LAL: 3.8 Leg agility left; 3.9 ACL: 3.9 Arising from chair lower limbs; period (.): missing data; A: Abdelwahab Elshourbagy; S: Samrah Javed; AS: Ahmed Omar Sadaney; H: Hassan Abdalshafy; J: James Robert Brašić; M: Mennatullah Mohamed Eltaras; %: Percentage agreement.

(McKay GN, Harrigan TP, Brasic JR. A low-cost quantitative continuous measurement of movements in the extremities of people with Parkinson's disease. MethodsX 2019; 6:169-189. <https://doi.org/10.1016/j.mex.2018.12.017>) [7].
